# Degradation of the low-calorie sugar substitute 5-ketofructose by different bacteria

**DOI:** 10.1007/s00253-021-11168-3

**Published:** 2021-02-22

**Authors:** Jacqueline Schiessl, Konrad Kosciow, Laura S. Garschagen, Juliane J. Hoffmann, Julia Heymuth, Thomas Franke, Uwe Deppenmeier

**Affiliations:** Institute of Microbiology and Biotechnology, University of Bonn, Meckenheimer Allee 168, 53115 Bonn, Germany

**Keywords:** Dietary sugar, Artificial sweetener, Low-calorie sugar, Ketofructose reductase, Human microbiota, Sugar-related disease

## Abstract

**Abstract:**

There is an increasing public awareness about the danger of dietary sugars with respect to their caloric contribution to the diet and the rise of overweight throughout the world. Therefore, low-calorie sugar substitutes are of high interest to replace sugar in foods and beverages. A promising alternative to natural sugars and artificial sweeteners is the fructose derivative 5-keto-D-fructose (5-KF), which is produced by several *Gluconobacter* species. A prerequisite before 5-KF can be used as a sweetener is to test whether the compound is degradable by microorganisms and whether it is metabolized by the human microbiota. We identified different environmental bacteria (*Tatumella morbirosei*, *Gluconobacter japonicus* LMG 26773, *Gluconobacter japonicus* LMG 1281, and *Clostridium pasteurianum*) that were able to grow with 5-KF as a substrate. Furthermore, *Gluconobacter oxydans* 621H could use 5-KF as a carbon and energy source in the stationary growth phase. The enzymes involved in the utilization of 5-KF were heterologously overproduced in *Escherichia coli*, purified and characterized. The enzymes were referred to as 5-KF reductases and belong to three unrelated enzymatic classes with highly different amino acid sequences, activities, and structural properties. Furthermore, we could show that 15 members of the most common and abundant intestinal bacteria cannot degrade 5-KF, indicating that this sugar derivative is not a suitable growth substrate for prokaryotes in the human intestine.

**Key points:**

• *Some environmental bacteria are able to use 5-KF as an energy and carbon source.*

• *Four 5-KF reductases were identified, belonging to three different protein families.*

• *Many gut bacteria cannot degrade 5-KF.*

**Supplementary Information:**

The online version contains supplementary material available at 10.1007/s00253-021-11168-3.

## Introduction

Several lines of evidence indicate that high sugar intake is a risk factor for the development of obesity, type 2 diabetes, and cardiovascular diseases (Malik et al. 2010; Schulze 2004). In addition, there is an increasing public awareness about the risk of dietary sugars (glucose, sucrose, and fructose) with respect to their caloric contribution to the diet and the rise of overweight of children and adults throughout the world (Schulze 2004; Ludwig et al. 2001). Therefore, low-calorie sugar substitutes are of high interest to replace sugar in foods and beverages. Based on the tendency of increased human sugar intake and the associated negative effects on human metabolism, the demand for alternative sweeteners has increased enormously. Especially naturally occurring low-calorie sweeteners have become the focus of the food industry. Among the already established synthetic sweeteners are saccharin, acesulfame-K, sucralose, aspartame, and neotame (Mattes and Popkin 2009). Disadvantages of these synthetically produced sweeteners can be, for example, expensive synthesis processes, artificial tastes, or undesirable side effects for consumers (Schiffman et al. 1979; Wiet and Beyts 1992). Two other non-artificial sugar substitutes used in the food industry are the ketohexose tagatose and the sugar alcohol xylitol. Although both compounds have a high degree of sweetening, they can be partially metabolized by the human metabolism and therefore have a caloric value (Grembecka 2015; Vastenavond et al. 2011). Alternative sweeteners of natural origin are steviol glycosides, which have 30 to 150 times the sweetness of sugar. However, the compounds may have a bitter or licorice-like aftertaste (Soejarto et al. 1982). A promising alternative to natural sugars and artificial sweeteners is the bacterially produced fructose derivative 5-keto-D-fructose (D-threohexo-2,5-diulose, 5-KF). 5-KF is a natural sugar produced by acetic acid bacteria through oxidation of fructose by the membrane-bound enzyme fructose dehydrogenase (Ameyama et al. 1981; Kawai et al. 2013). The compound has the same natural taste as fructose without an artificial aftertaste and a sweetness comparable to sucrose (Herweg et al. 2018). In addition, 5-KF cannot or can only partially be metabolized by the human organism and thus has a very low-calorie content (Wyrobnik et al. 2009). The new potential sweetener has already been detected in various natural foods, including white wine, honey, and elderflower syrup (Burroughs and Sparks 1973; Blasi et al. 2008).

5-KF is produced by the oxidation of fructose by some representatives of acetic acid bacteria of the genus *Gluconobacter.* Some of the organisms, such as *Gluconobacter* (*G.*) *japonicus*, possess the enzyme fructose dehydrogenase, which can oxidize fructose to 5-KF (Ameyama et al. 1981; Yamada et al. 1966). The genes coding for fructose dehydrogenase were introduced into *G. oxydans* 621H (Siemen et al. 2018) and the resulting genetically modified strain was capable of producing 5-KF in a fed-batch fermentation process resulting in product titers of up to 490 g L^−1^ and product yields up to 98% (Herweg et al. 2018). Furthermore, a *G. oxydans* strain was developed for the efficient production of 5-KF from the cost-efficient and renewable feedstock sucrose (Hoffmann et al. 2020). But before 5-KF can be used as a sweetener, it should be tested whether the compound is degradable by microorganisms and whether it is metabolized by the human microbiota. Therefore, we tested several bacteria for their ability to degrade 5-KF and we analyzed the enzymes involved in this process.

## Materials and methods

### Materials

All reagents, chemicals, and substrates used in this study were purchased from Carl Roth GmbH (Karlsruhe, Germany), Sigma-Aldrich (Taufkirchen, Germany), or Megazyme (Bray, Ireland). Q5 High-fidelity DNA polymerase, restriction endonucleases, T4 ligase, and PCR reagents were bought from Thermo Fisher Scientific Inc. (Waltham, USA) and New England Biolabs (Frankfurt am Main, Germany). Oligonucleotides were synthesized by Eurofins Scientific (Ebersberg, Germany).

### Culture conditions and standard molecular techniques

*G. oxydans* strains, *G. japonicus* LMG 26773, *G. japonicus* LMG 1281, and *Tatumella* (*T.*) *morbirosei* DSM 23827 (Table 1) were cultivated at 30 °C and 180 rpm. Precultures of *Gluconobacter* strains and *T. morbirosei* were grown in YM-medium (0.6% yeast extract, 0.9% mannitol) and in nutrient broth (0.5% peptone, 0.3% meat extract), respectively. For growth experiments, a medium consisting of 0.6% yeast extract was used. 5-KF was added as indicated. Precultures of *Clostridium* (*C.*) *pasteurianum* were cultivated anaerobically in a complex peptone yeast (PY) medium (Dowell and Hawkins 1974) containing 20 mM glucose in serum flasks under a N_2_/CO_2_ (80/20) atmosphere at 37 °C without agitation. Growth experiments with 5-KF were performed using a complex medium with 0.4% yeast extract, supplemented with 48 mM NaHCO_3_, 5% modified mineral 3B solution (40 mM KH_2_PO_4_, 300 mM NaCl, 4 mM CaCl_2_ × 2 H_2_O, 2 mM MgCl_2_ x 6 H_2_O, 1 mM MnCl_2_ × 4 H_2_O 0.1 mM CoCl_2_ × 6 H_2_O, 35 mM Na_2_SO_4_, (Varel and Bryant 1974)) 0.5 g L^–1^ cysteine and 25 mM substrate, pH 7, in serum flasks under N_2_ atmosphere. The pH was adjusted with NaHCO_3_. *Escherichia* (*E.*) *coli* DH5α was cultivated in lysogeny broth (LB) (Miller 1972) at 37 °C, 180 rpm. Ampicillin (100 μg mL^–1^) was added for plasmid maintenance.

**Table 1:** Strains, plasmids, and primers

Strain, plasmid, primer	Description	Source
Strain		
*Clostridium pasteurianum* DSM 525	Wild-type strain	DSMZ (Braunschweig, Germany)
*Escherichia coli* NEB® 5-alpha (referred to as *E. coli*)	F−, ø80d*lacZ*ΔM15, Δ(*lacZYA*-*argF*) U169, *deoR*, *recA*1, *endA*1, *hsdR*17 (rk−, mk+), *phoA*, *supE*44, λ−, *thi*-1, *gyr*A96, *rel*A1	New England Biolabs (Ipswich, USA)
*Escherichia coli* pASK5_KFR_TM	*E. coli* NEB® 5-alpha containing plasmid pASK5_KFR_TM	This study
*Escherichia coli* pASK5_gox0644	*E. coli* NEB® 5-alpha containing plasmid pASK5_ gox0644	(Schweiger et al. 2010)
*Escherichia coli* pASK.3_gox1432.ST	*E. coli* NEB® 5-alpha containing plasmid pASK.3_ gox1432.ST	(Zahid and Deppenmeier 2016)
*Escherichia coli* pASK5_AKR_Cpast	*E. coli* NEB® 5-alpha containing plasmid pASK5_ AKR_Cpast	This study
*Escherichia coli* pASK5_SDH_Cpast	*E. coli* NEB® 5-alpha containing plasmid pASK5_ SDH_Cpast	This study
*Escherichia coli* pASK5_gox1615	*E. coli* NEB® 5-alpha containing plasmid pASK5_ gox1615	(Schweiger et al. 2010)
*Escherichia coli* pASK5_gox2684	*E. coli* NEB® 5-alpha containing plasmid pASK5_ gox2684	(Schweiger et al. 2008)
*Escherichia coli* pASK5_gox0502	*E. coli* NEB® 5-alpha containing plasmid pASK5_ gox0502	(Schweiger et al. 2008)
*Escherichia coli* pASK5_gox0313	*E. coli* NEB® 5-alpha containing plasmid pASK5_ gox0313	(Schweiger et al. 2013)
*Escherichia coli* pASK3_gox0646	*E. coli* NEB® 5-alpha containing plasmid pASK3_ gox0646	(Schweiger et al. 2013)
*Escherichia coli* pASK5_gox1417	*E. coli* NEB® 5-alpha containing plasmid pASK5_ gox1417	(Rauch et al. 2010)
*Gluconobacter japonicus* LMG 1281	Wild-type strain; Cef^R^	BCCM
*Gluconobacter japonicus* LMG 26773	Wild-type strain; Cef^R^	BCCM
*Gluconobacter oxydans* 621H ∆*hsdR* (referred to as *G. oxydans*)	∆*hsdR (*∆*gox2567)* derivative of *G. oxydans* 621 H	S. Bringer-Meyer, Forschungszentrum Jülich GmbH
*Tatumella morbirosei* DSM 23827	Wild-type strain	DSMZ (Braunschweig, Germany)
Plasmids		
pASK-IBA.3	C-terminal Strep-tag II sequence, *tetA* promoter/repressor system, Amp^R^	IBA GmbH
pASK5-IBA.5	N-terminal Strep-tag II, *tetA* promoter/repressor system, Amp^R^	IBA GmbH
pASK5_KFR_TM	pASK-IBA.5 containing the gene *kfr* from *T. morbirosei* DSM 23827	This study
pASK5_gox0644	pASK-IBA.5 containing the gene *gox0644* from *G. oxydans* 621H ∆*hsdR*	(Schweiger et al. 2010)
pASK.3_gox1432.ST	pASK-IBA.3 containing the gene *gox1432* from *G. oxydans* 621H ∆*hsdR*	(Zahid and Deppenmeier 2016)
pASK5_AKR_Cpast	pASK-IBA.5 containing the gene *akr* from *C. pasteurianum* DSM 525	This study
pASK5_SDH_Cpast	pASK-IBA.5 containing the gene *sdh* from *C. pasteurianum* DSM 525	This study
pASK5_gox1615	pASK-IBA.5 containing the gene *gox1615* from *G. oxydans* 621H ∆*hsdR*	(Schweiger et al. 2010)
pASK5_gox2684	pASK-IBA.5 containing the gene *gox2684* from *G. oxydans* 621H ∆*hsdR*	(Schweiger et al. 2008)
pASK5_gox0502	pASK-IBA.5 containing the gene *gox0502* from *G. oxydans* 621H ∆*hsdR*	(Schweiger et al. 2008)
pASK5_gox0313	pASK-IBA.5 containing the gene *gox0313* from *G. oxydans* 621H ∆*hsdR*	(Schweiger et al. 2013)
pASK3_gox0646	pASK-IBA.3 containing the gene *gox0646* from *G. oxydans* 621H ∆*hsdR*	(Schweiger et al. 2013)
pASK5_gox0417	pASK-IBA.5 containing the gene *gox0417* from *G. oxydans* 621H ∆*hsdR*	(Rauch et al. 2010)
Primers	Primer sequence^a^	Restriction site
KFR_pASK5_for	ATTAGAATTCTGCTGAACAACAAAAT	*Eco*RI
KFR_pAKS5_rev	TAATCTCGAGTCAGCCGGTGAAAAGT	*Xho*I
AKR_Cpast_for	ATGGTAGGTCTCAGCGCGCCGTAAAATCTATTAAGGATGTTATAACTT	*Bsa*I
AKR_Cpast_rev	ATGGTAGGTCTCATATCACATCTGAA	*Bsa*I
SDH_Cpast_for	ATGGTAGGTCTCAGCGCCATGACATGTTTATTTGGACTTATAGG	*BsaI*
SDH_Cpast_rev	ATGGTAGGTCTCATATCATATTTGTTTTTTTAAAAATTTGTCATATATT	*BsaI*

^a^Restriction sites are underlined

### HPLC analysis

For the analysis of substrate consumption and product formation analysis, 1-ml samples were taken at different time points and centrifuged at 13,000×*g* for 1 min. The supernatants were diluted 1:10 with H_2_O. HPLC analysis (Knauer Smartline HPLC system, Knauer GmbH, Berlin, Germany) was performed using an Aminex HPX-87H 300 mm × 7.8 mm column (Biorad, Munich, Germany) with 5 mM H_2_SO_4_ at 65 °C and a flow rate of 0.6 ml min^–1^. Substrates and products were quantified by a refraction index detector (RI detector; Azura RID2.1 L, Knauer GmbH, Berlin, Germany) and a UV detector (Smartline 2600, Knauer, Berlin, Germany) at 210 nm by comparison to calibration curves. For product analysis of 5-KF reduction enzyme assays samples were analyzed by a SpectraSYSTEM HPLC system (Thermo Fisher Scientific Inc., Waltham, USA) using an amino phase column Eurospher II 100 NH2 (250 × 3.0 mm; 5 μm particle size) (Knauer GmbH, Berlin, Germany) with integrated precolumn, 90% acetonitrile as solvent, at 40 °C and a flow rate of 0.6 ml min^–1^. Products were quantified by a refraction index (RI) detector (Shodex RI-101) (Showa Denko Europe GmbH, Munich, Germany) and evaluated by the external standard method with ChromQuest 5.0 (Thermo Fisher Scientific Inc.).

### Construction of expression systems for *kfr* and *akr*

The gene *kfr* from *T. morbirosei* (HA49_09215) and *sdh* from *C. pasteurianum* (CPAST_c38270), both encoding a putative shikimate dehydrogenase, and the gene *akr* (CPAST_c22030), encoding an aldo/keto-reductase (AKR) from *C. pasteurianum*, were amplified by PCR using Q5 High-fidelity DNA polymerase and the primers KFR_pASK5_for/ KFR_pASK5_rev, SDH_Cpast_for/ SDH_Cpast_rev, and AKR_Cpast_for/ AKR_Cpast_rev, respectively (Table 1). As template genomic DNA of *T. morbirosei* DSM 23827 and genomic DNA of *C. pasteurianum* DSM 525 was used. The genes were cloned into a pASK-IBA.5 vector. The gene *kfr* from *T. morbirosei* was cloned via the restriction sites *Eco*RI and *Xho*I resulting in the plasmid construct pASK5_KFR_TM. The genes *sdh* and *akr* from *C. pasteurianum* were cloned via the restriction site *Bsa*I, resulting in the vector constructs pASK5_SDH_Cpast and pASK5_AKR_Cpast, respectively. The plasmids were transformed into competent *E. coli* DH5α cells by heat shock. Positive clones were verified by sequencing.

### Overexpression and purification of the proteins TM-KFR, CP-AKR, GOX0644, and GOX1432

For protein production, overnight cultures of *E. coli* (5 mL) harboring plasmids of interest were used to inoculate 1 L LB medium and were incubated at 37 °C and 180 rpm in shaker flasks. After reaching an OD_600_ of 0.4, protein production was induced by the addition of 0.2 μg mL^-1^ anhydrotetracycline and cells were further cultivated for 4 h. Cultures were harvested at OD_600_ between 1.0 and 1.5 by centrifugation (9000×*g*, 4 °C, 15 min). Lysis and purification were performed by sonication as previously described (Kosciow et al. 2016). For protein visualization, polyacrylamide gel electrophoresis was done according to (Laemmli 1970) and protein bands were detected via silver stain as described by (Blum et al. 1987). Analysis of the native conformation of the ketofructose reductases was performed by gel filtration chromatography using a HiLoad 16/60 Superdex 200 pg column (GE Healthcare, Chicago, USA) connected to an ÄKTApurifier system (GE Healthcare, Chicago, USA). The column was calibrated using the Gel Filtration Calibration Kit HMW (GE Healthcare, Chicago, USA). Equilibration was done with 50 mM Tris-HCl buffer pH 8, containing 150 mM NaCl.

### Measurement of enzyme activities and kinetic parameters

The reduction of 5-KF and other substrates accompanied by the oxidation of NAD(P)H to NAD(P)^+^ was recorded at 340 nm (ɛ=6.22 mM^−1^cm^−1^). The reaction mixture with a final volume of 1 ml contained 250 μM NAD(P)H, 40 mM potassium phosphate buffer (pH 7), and varying substrate concentrations between 5 and 20 mM. One unit of enzyme activity corresponded to the oxidation of 1.0 μmol of substrate per min and mg protein. The pH optima were determined using the McIlvaine buffer system (McIlvaine 1921) between pH 3.0 and 9.0 containing disodium phosphate and citric acid. Temperature optima were analyzed in a temperature range between 20 °C and 80 °C using standard assay conditions. Nonlinear regressions of Michaelis–Menten data were used to calculate kinetic constants at optimal pH and temperate using 5-KF concentrations between 0.5 and 80 mM and NAD(P)H concentrations between 5 μM and 250 μM, respectively.

Enzyme assays analyzed via HPLC were performed with the same principle as described above. The enzyme assays had a final volume of 250 μL with 5 mM NADPH, 5 mM 5-KF, 40 mM potassium phosphate buffer pH 7 or pH 6 for CP-AKR and 2 μg enzyme. The reaction mixture was incubated at 30 °C or 40 °C for CP-AKR for three hours. The enzyme reaction assays were directly analyzed *via* HPLC, as described above.

### Transcriptional analysis by qRT-PCR experiments

Total RNA from *T. morbirosei*, *C. pasteurianum*, or *G. japonicus* LMG 23776 was isolated from 50 ml cultures grown to mid-exponential phase as described by Kröninger et al. (2017). qRT-PCR reactions were performed with a CAPITAL^TM^ qRT-PCR Green Mix One Step Kit (Biotechrabbit, Hennigsdorf, Germany), which uses SYBR Green as the fluorescent dye. Each PCR reaction contained 150-200 ng of purified RNA. Temperature cycling and fluorescence measurement was performed with a cycler CFX Connect™ and suitable software (BioRad, Munich, Germany). The ΔCt values were calculated by subtraction of the quantification cycle (Ct) of the reference gene (encoding the 50S ribosomal protein L10) from the Ct value of each gene of interest. The relative change in mRNA abundance was recorded as the ratio of normalized target concentrations (ΔΔCt). The fold change of each gene was calculated using the formula 2^−ΔΔCt^. The following genes were analyzed: *T. morbirosei* gene HA49_09215 encoding protein KGD75378 (TM-KFR; primers KFR_fw: CGGCGACCTATGTTGAATGG and KFR_rev: GGAAGCAGCACGTAACAG AG), *T. morbirosei* gene HA49_21310 encoding the 50S ribosomal protein L10 as reference gene (primers T.morb_rib50S_fw: TCGTGAAGCTGGCGTATACA and T.morb_rib50S_rev: TTTGAACAGACGAG CAGCAG), *C. pasteurianum* gene CPAST_c22030 encoding protein AJA48273.1 (CP-AKR; primers C.past_AKR_fw: CGGAAGGGGTGCTGTTCTTA and C.past_AKR_rev: TCCCCAGTTCTTTCAGCTC T), *C. pasteurianum* gene CLPA_c36850 encoding the 50S ribosomal protein L10 as reference gene (primers: C.past_rib50S_fw: CGTGCTGCAAATGAACTTGG and C.past_rib50S_rev: CGCCTTGTA CTAATCCAGCC), *G. japonicus* LMG 26773 gene AD942_05425 encoding protein KXV40663 (GJA0644; primers G.jap-1-_KFR_fw: GCACTTGTTGACCTGCAGAA and G.jap-1-_KFR_rev: TGCGGATGTTATGCTGTTCG), *G. japonicus* LMG 26773 gene AD942_03970 encoding protein KXV41067 (GAJ1432; primers: G.jap-2-_KFR_fw: GATCTGAAGACGCCGGAAAC and G.jap-2-_KFR_rev: GATGCGATCAACCATGCCAT), and *G. japonicus* LMG 26773 gene AD935_10055 encoding the 50S ribosomal protein L10 as reference gene (primers: G.jap_rib50S_fw: AGAACAAGGGTCTGACGGTT and G.jap_rib50S_rev: AGCTTGTCATTCGTCTTGGC).

### Bioinformatic tools

The program Blastp at the NCBI database (https://blast.ncbi.nlm.nih.gov/Blast.cgi) was used to identify proteins and to compare amino acid sequences (Johnson et al. 2008). The term similarity is defined as percentage of the number of amino acids that were either identical between the query and the subject sequence or had similar chemical properties. Amino acid sequence alignments were performed with the program Clustal Omega (https://www.ebi.ac.uk/Tools/msa/clustalo/) using default parameters. Kinetic parameters of purified enzymes were determined using nonlinear regression of the Michaelis–Menten data with the program GraphPad Prism Version 9.0.0

## Results

### 5-KF consumption in aerobic bacteria

**Fig. 1:**
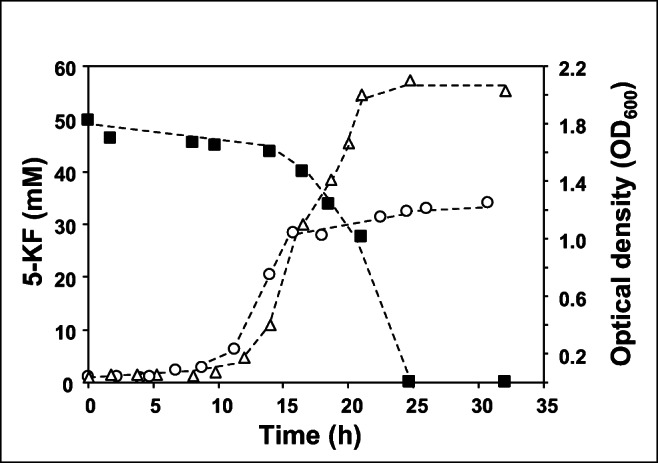
Growth and 5-KF consumption in *T. morbirosei.* Cells were grown in 0.6% yeast extract with 50 mM 5-KF or without 5-KF at 30 ^o^C. (■) 5-KF concentration, (Δ) OD_600_ of 5-KF containing cultures, (○) OD_600_ of control cultures without 5-KF. The figure shows a representative experiment from three biological replicates (for additional experiments see Fig. S1)

It was already shown that *T. citrea* (former *Erwinia citreus*) is capable of reducing 5-KF. The organism possesses a 5-KF reductase, which catalyzes the reversible NADPH-dependent reduction of 5-KF to D-fructose (Schrimsher et al. 1988). We tested a close relative, *T. morbirosei*, for its ability to grow on 5-KF as a carbon and energy source. A complex medium was used for the growth experiments since no mineral medium is available for *T. morbirosei* and the growth requirements of this organism are unknown. In the control experiment without the addition of a sugar source, the cells grew to an optical density (OD_600_) of 1.25 within 20 h (Fig. 1). The doubling time (t_d_) was 2.3 h. Cultures with 5-KF revealed a similar growth pattern at the beginning of the experiment (t_d_ = 1.8 h) with a short slowdown of growth after 16 h. In contrast to the negative control, a second growth was observed and the OD_600_ increased to 2.1 (Fig. 1), indicating a typical pattern of diauxic growth. In line with this observation was the fact that the organism consumed significant amounts of 5KF only after 16 h. 5-KF was completely utilized after 25 h and cells turned into the stationary growth phase. It can therefore be assumed that components of the complex medium were first used for growth, as in the control without 5-KF. Only in the second phase, 5-KF was utilized, which was accompanied by an increased OD_600_. *T. morbirosei* was therefore able to use 5-KF as a substrate.

**Fig. 2:**
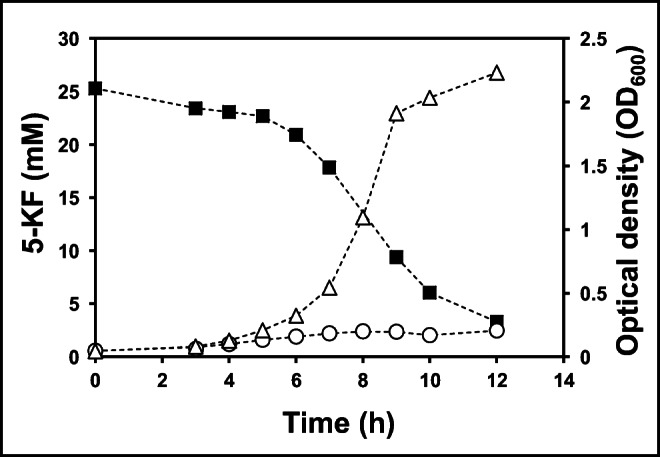
Growth of *G. japonicus* LMG 26773*.* Cells were grown in 0.6% yeast extract with 25 mM 5-KF or without 5-KF at 30 °C. (■) 5-KF concentration, (Δ) OD_600_ in the presence of 5-KF, (○) OD_600_ in the absence of 5-KF. One out of three independent experiments is shown (for additional information, see Fig. S2)

Some species of the genus *Gluconobacter* are known to produce 5-KF from fructose. The membrane-bound fructose dehydrogenase (Fdh), which is encoded by the *fdhSCL* genes, plays a crucial role in this process (Kawai et al. 2013). The enzyme oxidizes fructose at the hydroxyl group at position 5, thus forming 5-KF and transferring the released electrons into the respiratory chain of the organism. The resulting 5-KF diffuses via porins in the outer membrane into the extracellular space and accumulates in the culture supernatant. Hence, the question arose whether 5-KF can also be a substrate for growth. For this purpose, the Fdh-producing strain *G. japonicus* LMG 26773 was tested, which is able to form 5-KF. The organism was grown on a medium containing 0.6% yeast extract and 25 mM 5-KF (Fig. 2). It was found that *G. japonicus* LMG 26773 consumed large quantities of 5-KF after a lag phase of about 3 h and almost completely converted the substrate after 12 h. The OD_600_ increased from 0.05 to 2.2 within 9 h and the doubling time was 1.3 h. The control culture without 5-KF addition showed only an increase in OD_600_ from 0.05 to 0.2 (Fig. 2).

**Fig. 3:**
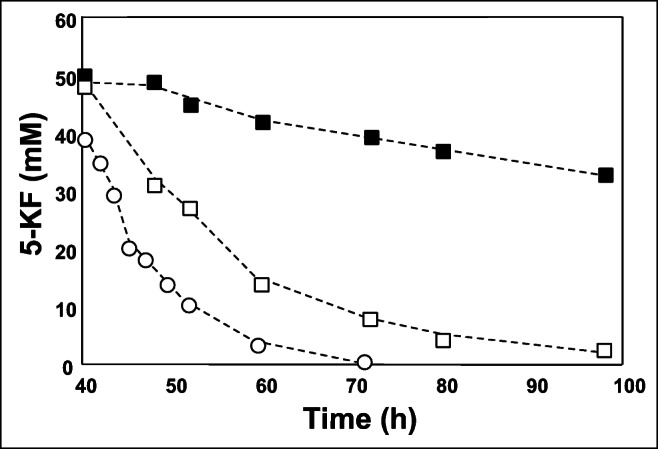
Degradation of 5-KF in the stationary growth phase by different *Gluconobacter* strains grown on 0.6% yeast extract and fructose as substrate. (■) *G. oxydans fdh*, (□) *G. japonicus* LMG 1281, (○) *G. japonicus* LMG 26773. The experiment was conducted in triplicate. 5-KF was added to a final concentration of 50 mM. The figure shows a representative experiment from three biological replicates (additional experiments are shown in Fig. S3)

The model organism *G. oxydans* 621H does not possess the key enzyme Fdh for 5-KF production (Siemen et al. 2018). Therefore, we tested a *fdh* knock-in mutant of *G. oxydans* (Hoffmann et al. 2020) for the expression of the *fdh* genes that allowed the production of the fructose dehydrogenase. Two other strains *G. japonicus* LMG 1281 and *G. japonicus* LMG 26773 naturally contained the *fdh* genes on their chromosomes (gene no. BAM93250 - BAM93252 and KXV40773 - KXV40775, respectively). As expected, all strains grew with fructose as substrate and accumulated 5-KF in the culture supernatant up to a concentration of 50 mM (Hoffmann et al. 2020). In the stationary phase, *G. japonicus* LMG 1281 and *G. japonicus* LMG 26773 completely consumed 5-KF within the period 40–98 h and 40–70 h, respectively (Fig. 3). In *G. oxydans fdh* the degradation of 5-KF was slow and only 10 mM 5-KF were utilized within 60 h.

### 5-KF consumption in anaerobic bacteria

**Fig. 4:**
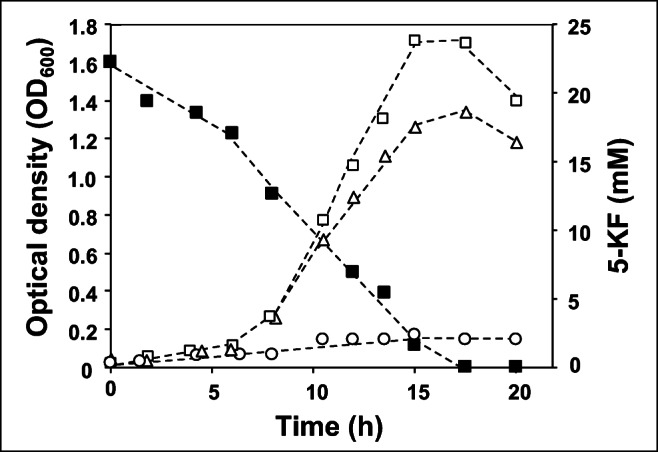
Growth and 5-KF consumption in *C. pasteurianum.* Cells were grown at 37 °C in 0.4% yeast extract medium supplemented with 48 mM NaHCO_3_, modified mineral 3B solution, and 23 mM 5-KF or 23 mM glucose. (■) 5-KF concentration, (Δ) OD_600_ of 5-KF containing cultures, (○) OD_600_ of control cultures without carbon source, (□) OD_600_ of glucose grown cultures. One out of three independent experiments is shown (the other experiments are shown in Fig. S4)

All 5-KF degrading species described above were strict aerobes and needed O_2_ as the final acceptor of their respiratory chain. Hence, the question arose whether only aerobic bacteria are able to ferment 5-KF or whether this ability is also observed in anaerobic bacteria. As a model organism we tested *C. pasteurianum* DSM 525 for its ability to use 5-KF as a substrate. The organism reached an OD_600_ of 1.7 and a doubling time of 2.0 h with 25 mM glucose as its preferred carbon source. In contrast, *C. pasteurianum* grew to a final OD_600_ of 1.4 within 17.5 h in the presence of 23 mM 5-KF, which corresponded to a growth rate of 0.33 h^-1^ and a doubling time of 2.1 h in the exponential phase (Fig. 4). HPLC analysis revealed a complete consumption of 5-KF after 17.5 h and the synthesis of the expected end products butyrate and acetate, indicating that this compound was metabolized by *C. pasteurianum.* Without an additional carbon source, *C. pasteurianum* reached only a final OD_600_ of 0.15 (Fig. 4).

Since we could show that the anaerobic bacterium *C. pasteurianum* is able to metabolize 5-KF, the next step was the investigation of whether the main players of the anaerobic human gut microbiota also have the ability to use 5-KF as a carbon and energy source. For consumers and the food industry, these experiments are important as they allow the determination of the effective caloric value of novel and potential sweeteners. Thus, 15 microorganisms were tested as representatives of important members of the human gut microbiota: *Agathobacter rectalis* DSM 17629, *Akkermansia muciniphila* DSM 22959, *Alistipes shahii* DSM 19121, *Bacteroides vulgatus* DSM 1447, *Bifidobacterium adolescentis* DSM 20083, *Clostridium leptum* DSM 753, *Collinsella intestinalis* DSM 13280, *Dorea formicigenerans* DSM 3992*, E. coli* K12, *Eubacterium siraeum* DSM 15702, *Lactobacillus reuteri* DSM 17509, *Methanomassiliicoccus luminyensis* DSM 25720, *Parabacteroides johnsonii* DSM 18315, *Prevotella copri* DSM 18205 and *Ruminococcus gnavus* DSM 108212 (Tab. S1). However, it became evident that the tested organisms did not reach a higher OD_600_ with 20 mM 5-KF as substrate when compared to the negative control without an additional carbon source (Fig. S5). Furthermore, HPLC analysis confirmed that 5-KF was not degraded by these bacteria. The results indicated that many important microbial species within the human gut are likely not able to metabolize 5-KF and to produce short-chain fatty acids, which could contribute to the total calorie intake of the human body.

### Characterization of 5-KF reductases

**Fig. 5:**
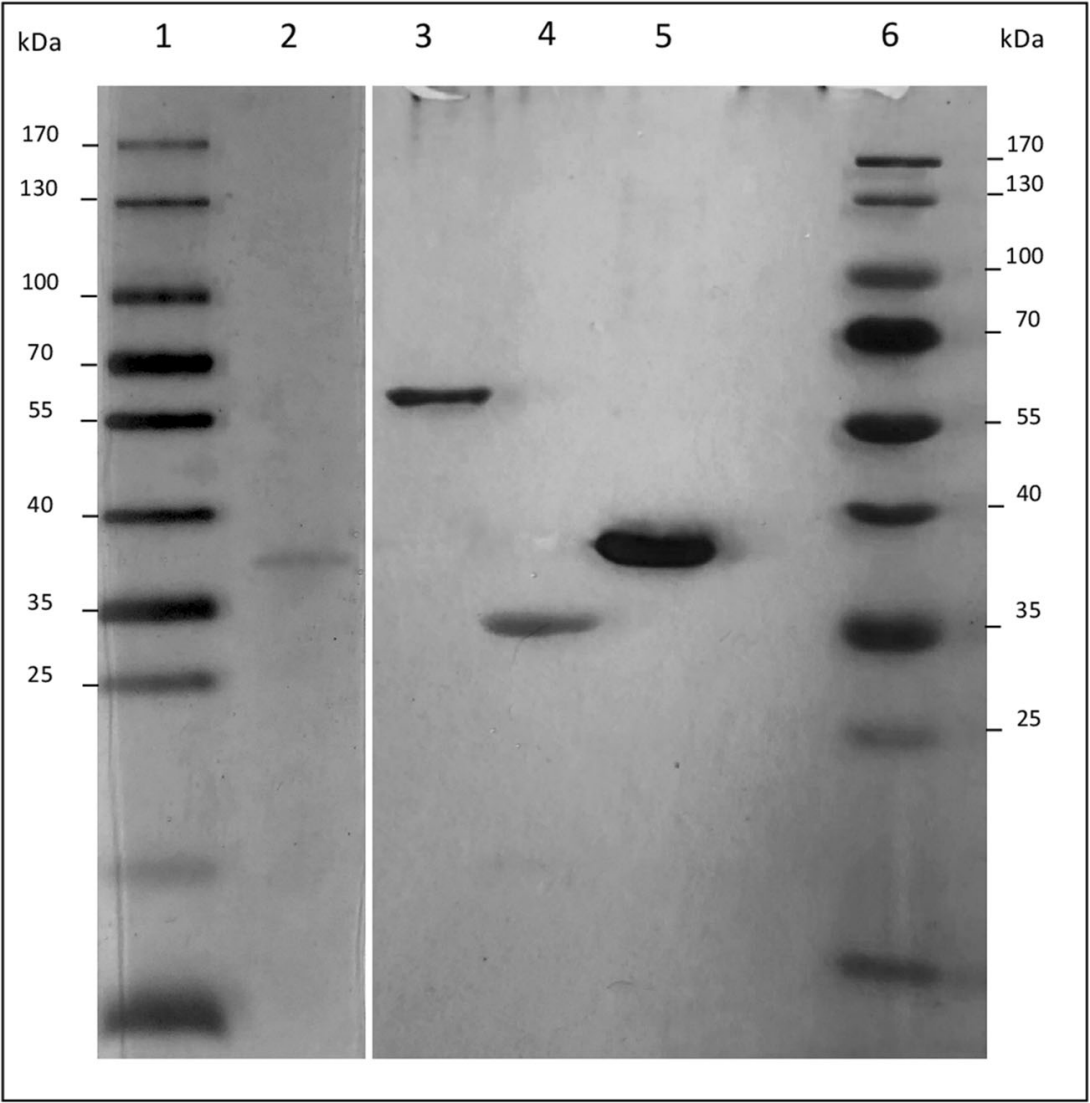
SDS-PAGE analysis of reductases. Lane 1 and 6: PageRuler™ Prestained Protein Ladder (Thermo Fisher Scientific), Lane 2: CP-AKR (1 μg protein). Lane 3: GOX1432 (2 μg protein). Lane 4: GOX0644 (2 μg protein). Lane 5: TM-KFR (4 μg protein)

As shown above, a couple of bacteria can grow with 5-KF as carbon and energy source. Others were able to use the compound in the stationary phase to ensure survival in their substrate-limited habitat. However, the knowledge about biochemical reactions that are involved in 5-KF degradation is limited. It is known that *T. citrea* contains a 5-KF reductase that can reduce 5-KF using NADPH as reductant. The corresponding enzyme was purified and characterized (Schrimsher et al. 1988). The N-terminal AS sequence was published and a BLASTx search (https://blast.ncbi.nlm.nih.gov/Blast.cgi) resulted in the identification of protein from *T. morbirosei* with 94% similarity. Hence, we could identify the corresponding gene (HA49_09215) and amino acid sequence (KGD75378.1). The corresponding DNA fragment was amplified by PCR and the gene was cloned into plasmid pASK-IBA.5, which allowed the precise fusion with the vector encoded Strep-tag II sequence. The protein was produced in *E. coli* and purified to apparent homogeneity with the help of a streptavidin affinity column (Fig. 5). The protein was referred to as TM-KFR.

As demonstrated by SDS-PAGE (Fig. 5), TM-KFR showed the expected protein band with a molecular mass of 38 kDa. The native protein revealed a single active peak when analyzed by gel filtration. The peak corresponded to 121.3 kDa, indicating that the enzyme is active as a trimer (Table 2). The apparent K_M_ value of the enzyme for 5-KF, estimated by using several concentrations of the substrate from 0.5 to 40 mM, was 6.4 ± 0.6 mM at pH 7.0 and 30 °C (Table 2), while V_max_ was 1522.8 ± 141.9 U mg^-1^. The apparent K_M_ value for NADPH was 20.4 ± 11.5 μM. For a detailed analysis of enzymatic activities, we first determined the substrate spectrum of the protein. It became evident that the enzyme only reduced 5-KF and could only utilize NADPH as cofactor. All other compounds tested (aldehydes, ketones, sugars, keto sugars, sugar acids, polyols, Table 3) were not attacked. Hence, the specificity towards 5-KF can be used to determine the presence and concentration of the alternative sweetener in various foods and beverages in a photometric assay. Such tests were performed and it was found that 5-KF is present in different white wines, honey, and vinegar in micromolar concentrations (not shown).

**Table 2:** Properties of 5-KF reducing enzymes

Organism	Enzyme	Monomer [kDa]^1^	Conformation	Temp.-optimum [°C]	pH- Optimum	Specific activity [U/mg]	K_cat_ [s^-1^]	K_M 5-KF_ [mM]	K_M NADPH_ [μM]
*T. morbirosei*	TM-KFR	36.0	trimer	30	7	1522.8±141.9	913.9 ± 85.2	6.4±0.6	20.4±11.5
*G. oxydans*	GOX0644	34.6	dimer	35	7	32.7±2.1	18.9 ± 1.2	4.0±0.8	61.2±11.4^2^
*G. oxydans*	GOX1432	55.9	monomer	30	7	230.3±8.0	214.6 ± 7.5	42.0±5.4	19.5± 1.0^3^
*C. pasteurianum*	CP-AKR	35.9	tetramer	40	6	48.9±7.1	29.3 ± 4.3	1.3±0.2	12.4±3.8

^1^Molecular mass including Strep-tag

^2^Schweiger et al. 2010

^3^Zahid and Deppenmeier 2016

**Table 3:** Analysis of the substrate spectrum of 5-KF reductases

		Specific activity [U mg^-1^]			
	Substrates^1^	TM-KFR	GOX0644	CP-AKR	GOX1432
Ketones	2-butanol-3-one	0	0	0.2	0
	1-phenyl-1,2-propandione	0	71.2	5.3	0
	pentane-2,3-dione	0	20	0	0
Aldehydes	diacetyl	0	14.3	13.5	0
	D-/L-glyceraldehyde	0	0.1	0	0
	oxalaldehyde	0	1.1	0.4	0
	pyruvaldehyde	0	10.8	1.9	0
Ketosugars	5-ketofructose	1428	29.5	35.6	130
	5-ketogluconate	0	0	0.1	0.6
	L-sorbosone	0	0	0.2	0
Sugars	D-fructose	0	0	0	17.2
	L-sorbose	0	0	0	6.8

^1^Enzyme assays were performed at 30 °C in 1 ml assays containing 40 mM potassium phosphate buffer pH 7 or pH 6 for CP-AKR, 250 μM NADPH and 20 mM substrate as indicated. None of the enzymes could reduce D-arabinose, L-arabinose, glucose, mannose, ribose, tagatose, xylose, maltose, raffinose, sucrose, trehalose, and xylobiose

**Fig. 6:**
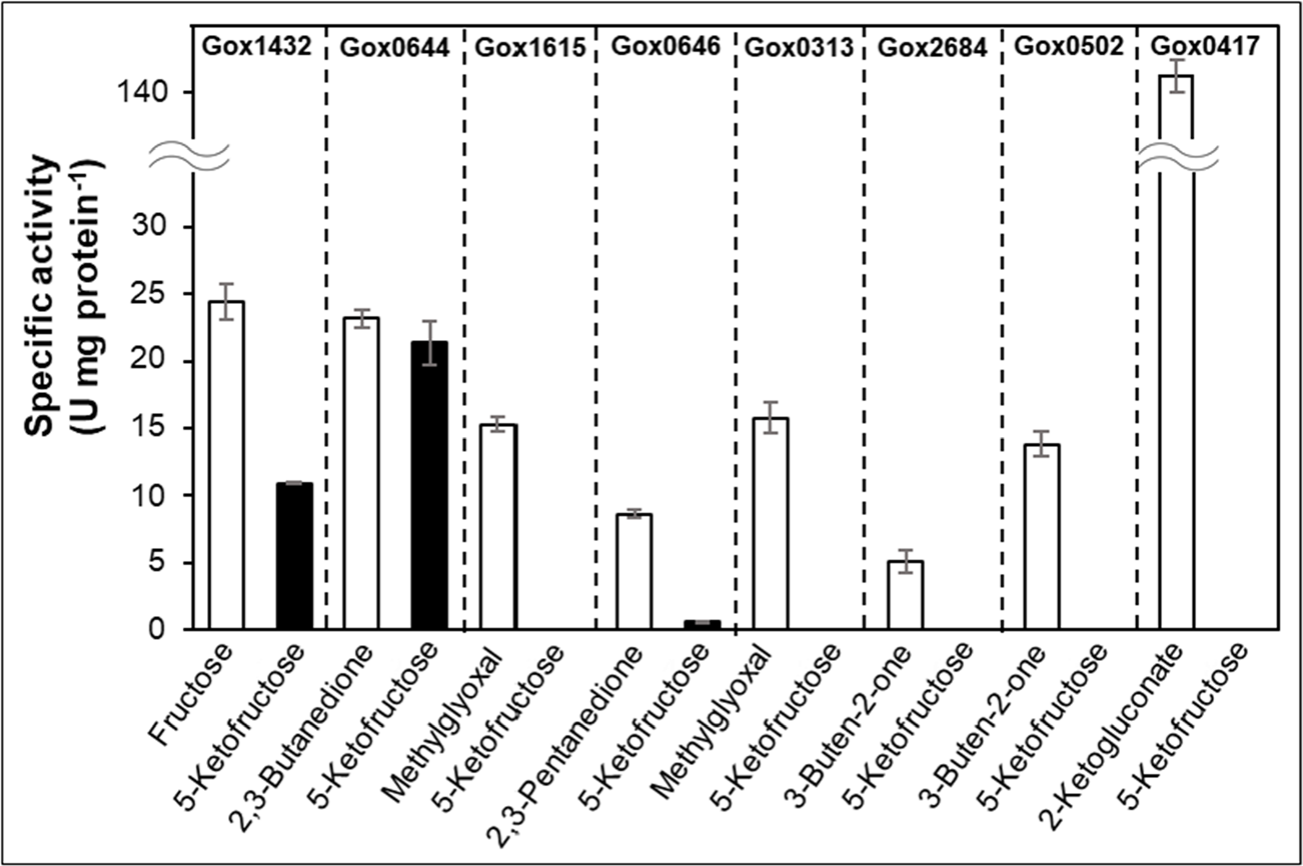
Analysis of oxidoreductases from *G. oxydans*. Activity with known substrates (white columns), activity with 5-KF (black columns). Assay consisted of 40 mM potassium phosphate buffer pH 7, 250 μM NADPH, 5 mM substrate and 2 μg enzyme. The assays were incubated at 30 °C

As shown above, *G. oxydans* was not able to use 5-KF as carbon and energy source in the exponential growth phase, but consumption of 5-KF was observed in the stationary phase (Fig. 3). To verify which enzymes are responsible for this effect, we tested several oxidoreductases that act on organic compounds containing keto groups for their ability to reduce 5-KF to fructose. All enzymes indicated in Fig. 6 were heterologously produced in *E. coli* and purified by Strep-tag affinity chromatography. It became evident that the 5-ketogluconate reducing enzyme GOX1432 (Zahid and Deppenmeier 2016) and the α-ketocarbonyl reductase GOX0644 (Schweiger et al. 2010) were able to reduce 5-KF with NADPH as reductant (Fig. 6). The α-diketone reductase GOX0646 (Schweiger et al. 2013) reduced 5-KF at a very low rate. In contrast, the 2-ketogluconate reductase GOX0417 (Rauch et al. 2010) and the vinyl ketone reductases GOX0502 and GOX2684 (Schweiger et al. 2008) did not reduce 5-KF. The same was true for the α-ketocarbonyl reductases GOX1615 (Schweiger et al. 2010) and GOX0313 (Schweiger et al. 2013) (Fig. 6).

SDS-PAGE analysis showed molecular masses of 34 kDa and 55 kDa for the proteins GOX0644 and GOX1432, respectively (Fig. 5), which were in good correlation with the estimated masses predicted from their amino acid sequences. The native conformation of both enzymes was analyzed via gel filtration, indicating a monomeric structure for protein GOX1432 and a dimeric conformation for protein GOX0644. Kinetic properties of the enzymes with 5-KF as substrate were examined with nonlinear regressions of Michaelis–Menten data. The K_M_ and V_max_ values of protein GOX0644 for 5-KF as substrate were 4.0 ± 0.8 mM and 32.7 ± 2.1 U mg^–1^ (Table 2). In case of protein GOX1432, a V_max_ value of 230.3 ± 8.0 U mg^–1^ was calculated, which indicates a higher specific activity for 5-KF when compared to GOX0644. In contrast, protein GOX1432 showed a significantly lower affinity for this substrate (K_M_ value: 42.0 ± 5.4 mM; Table 2). With NADPH as co-substrate of the reaction, K_M_ values of 61.2 ± 11.4 μM and 19.5 ± 1.0 μM were determined, respectively. The substrate spectra of the enzymes were already published. Protein GOX0644 reduced α-ketoaldehydes, α-diketones, α-keto esters (Table 3), and the sugar derivative 2,5-diketogluconate (Schweiger et al. 2010), which shows structural homologies to the newly discovered substrate 5-KF. Protein GOX1432 was previously described as mannitol dehydrogenase and reduced the sugars D-fructose and L-sorbose as well as the keto sugar 5-keto-D-gluconate (Table 3) (Zahid and Deppenmeier 2016).

As demonstrated above, *C. pasteurianum* was able to grow on 5-KF. Since the three proteins KFR from *T. morbirosei*, GOX0644 and GOX1432 from *G. oxydans*, were characterized as 5-KF reducing enzymes, Blastp analyses (https://blast.ncbi.nlm.nih.gov/Blast.cgi) were performed to identify corresponding enzymes in *C. pasteurianum*. Two proteins encoding a putative shikimate dehydrogenase (AJA49865.1) and a putative aldo/keto reductase (AJA48273.1) were similar to the KFR from *T. morbirosei* and protein GOX0644, respectively (similarities of 48% and 67%). Both enzymes were produced in *E. coli* and purified via streptavidin affinity chromatography. While the putative shikimate dehydrogenase AJA49865.1 was inactive with 5-KF as substrate, high activity was detected for the aldo/keto reductase AJA48273.1 (referred to as CP-AKR). When analyzed by polyacrylamide gel electrophoresis and silver stain, a single band for CP-AKR could be detected that was in good accordance with the expected size of 35 kDa (Fig. 5). Gel filtration analysis revealed that the native enzyme formed homotetramers. In addition, the kinetic parameters were investigated, indicating a V_max_ value for 5-KF of 48.9 ± 7.1 U mg^–1^ and a low K_M_ value of 1.3 ± 0.2 mM. The K_M_ for NADPH was 12.4 ± 3.8 μM. The CP-AKR was able to reduce aldehydes as well as different ketones and, additionally, the reduction of 5-ketogluconate and L-sorboson could be shown (Table 3).

**Fig. 7:**
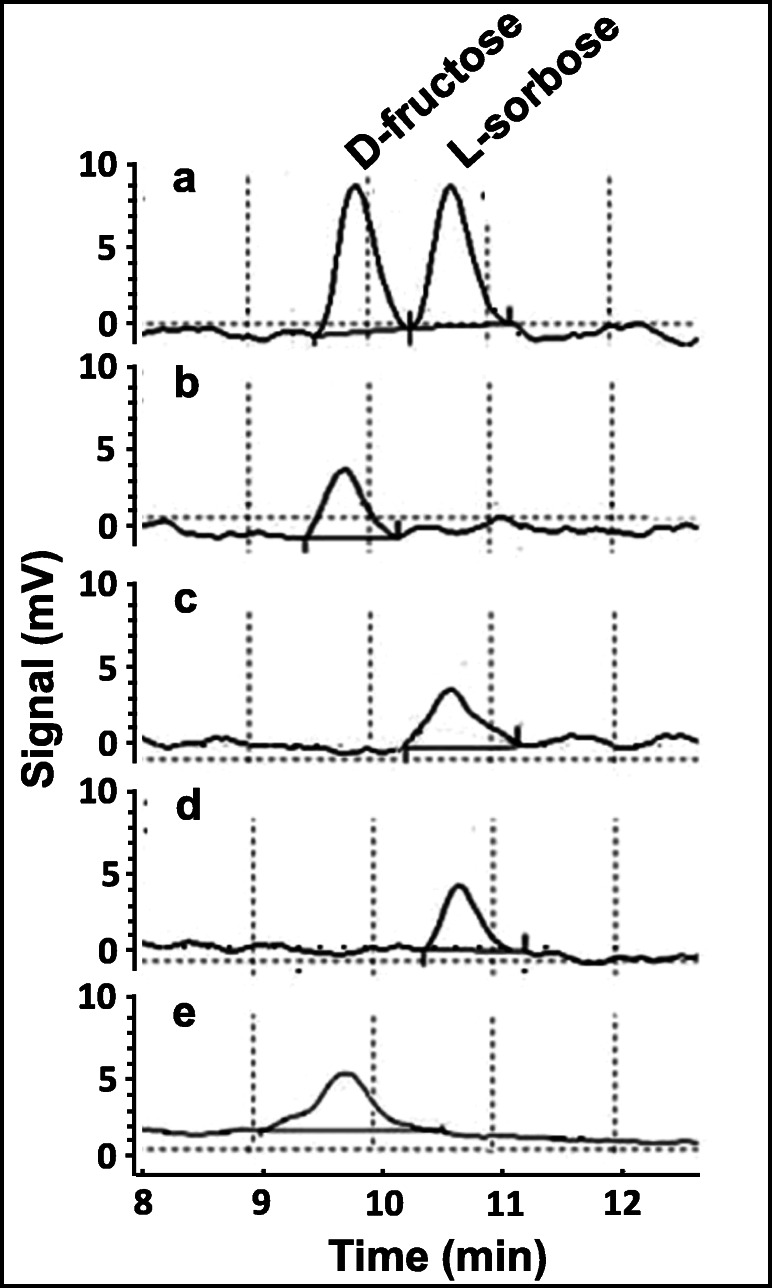
Product analysis of 5-KF reduction. **a** D-fructose and L-sorbose standards. **b** Product of TM-KFR. **c** Product of protein GOX0644. **d** Product of CP-AKR. **e** Product of protein GOX1432. Enzyme assays contained 40 mM KP buffer pH 7 or pH 6 for CP-AKR, 5 mM NADPH, 5 mM 5-KF and 2 μg enzyme. The assays were incubated at 30 °C or at 40 °C for CP-AKR for 3 h. Reaction products were quantified by HPLC analysis via a UV detector

Although all described enzymes exhibited 5-KF reductase activity, significant differences were determined concerning the substrate spectrum, the corresponding amino acid sequence, and the tertiary and quaternary structure. To test whether also the reaction mechanism is different, an HPLC analysis of the end products of all four enzymes after 5-KF reduction was performed (Fig. 7). D-fructose and L-sorbose were applied as analytical standards (Fig. 7a). It became evident that protein GOX0644 and its homologous protein CP-AKR produced L-sorbose as main product of 5-KF reduction (Fig. 7 c and d). In contrast, protein GOX1432 and TM-KFR from *T. morbirosei*, which did not show sequence similarities, reduced 5-KF to D-fructose (Fig. 7b and e). A closer look at the structure of 5-KF indicates that both prochiral keto groups are homotopic and have therefore the same chemical reactivity. Each sp^2^-hydridized trigonal planar carbonyl C-atom can be reduced by the enzymes from the *re* or the *si* face. Hence, the stereoselectivity of the 5-KF reducing enzymes with respect to the hydrid transfer from dehydronicotinamide to the substrate is responsible for the formation of D-fructose or L-sorbose.

### Involvement of the purified enzymes in the metabolism of 5-KF degrading bacteria

**Fig. 8:**
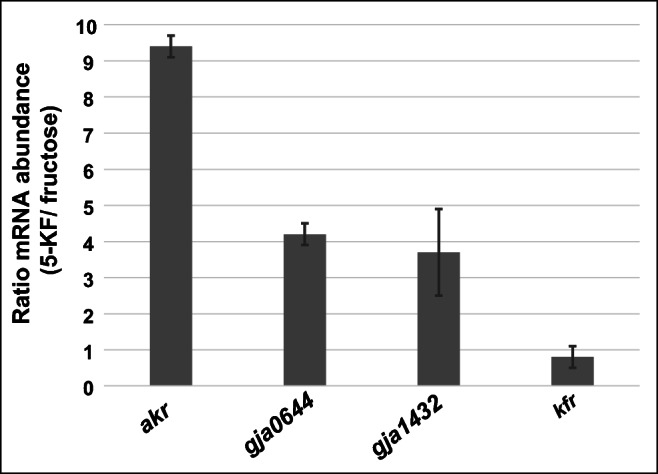
Ratio of mRNA abundance of genes encoding 5-KF reducing enzymes. ΔCt values were calculated using the amount of ribosomal protein L21 transcripts as reference. Ratios were calculated from ΔΔCt values using the function 2^–ΔΔCt^. Experiments were performed with RNA preparation from three different cultures harvested in the mid-exponential growth phase. Analyzed genes: *akr*, gene CPAST_c38270 encoding the 5-KF reducing aldo/keto reductase CP-AKR (AJA49865.1) from *C. pasteurianum* DSM 525; gja0644, gene AD942_05425 encoding the 5-KF reducing enzyme GJA0644 (KXV40663.1) from *G. japonicus* LMG 26773; gja1432, gene AD942_03970 encoding the 5-KF reducing enzyme GJA1432 (KXV41067.1) from *G. japonicus* LMG 26773; kfr, gene HA49_09215 encoding the 5-KF reductase (KGD75378.1) from *T. morbirosei* DSM 23827

*C. pasteurianum* was able to grow with 5-KF (Fig. 4). Furthermore, the aldo/keto reductase CP-AKR (AJA48273.1) from *C. pasteurianum* showed high 5-KF reducing activity. qRT-PCR experiments revealed that the mRNA abundance of the corresponding gene was increased about 10-fold during growth with 5-KF (Fig. 8), indicating that CP-AKR had an important function in the degradation of this substrate and might be responsible for the formation of fructose, which is then channelled into the central metabolism of *C. pasteurianum*. *G. oxydans* did not use 5-KF as substrate for growth. Therefore, it was not possible to test whether the proteins GOX0644 and GOX1432 were responsible for 5-KF reduction in vivo. However, in the 5-KF degrading bacterium *G. japonicus* LMG 26773, proteins with high similarity to the 5-KF reducing enzymes GOX0644 and GOX1432 were identified (Fig. S6). These enzymes were referred to as GJA0644 and GJA1432. The corresponding genes were analyzed by qRT-PCR and it was found that the mRNA concentrations were about 4-fold higher in 5-KF grown cells compared to cells, which used fructose as substrate (Fig. 8). The results indicate that the proteins GJA0644 and GJA1432 as counterparts of GOX0644 and GOX1432 are most probably involved in the first step of 5-KF degradation as performed by *G. japonicus* LMG 26773. In contrast to *C. pasteurianum* and *G. japonicus* LMG 26773, a change in the expression pattern of the gene encoding the 5-KF reductase TM-KFR was not observed in *T. morbirosei* (Fig. 8). However, for *T. citrea*, an almost identical 5-KF reductase has already been described and the enzyme was directly connected with the conversion of 5-KF into fructose as substrate for growth (Schrimsher et al. 1988).

## Discussion

In recent years, it has been demonstrated that conventional sugars such as sucrose, glucose, and fructose can promote the development of food-related diseases (Malik et al. 2010; Schulze 2004; Ludwig et al. 2001; Malik et al. 2006; Johnson et al. 2007) such as type 2 diabetes, obesity, and cardiovascular diseases. It can also be observed that consumers are increasingly paying attention to a more conscious diet leading to an increased demand for calorie-free or low-calorie substitutes as an alternative to traditional sweeteners (Marti et al. 2008; Ogden et al. 2006). A promising alternative to natural sugars and artificial sweeteners is the bacterially produced fructose derivative 5-KF. Tests with trained taste specialists have shown that the sweetening power and taste are equivalent to the properties of pure fructose. Thus, 5-KF has a stronger sweetening power than sucrose (Herweg et al. 2018; Stone and Oliver 1969). The compound revealed a similar intrinsic sweet threshold concentration in comparison to fructose. Moreover, there is no artificial, bitter or licorice-like aftertaste, which is noticeable when using some other alternative sweeteners such as stevia, saccharin, and acesulfame K (Soejarto et al. 1982; Herweg et al. 2018; Kuhn et al. 2004). In addition, 5-KF is a natural sugar derivative that is found, e.g., in honey, white wine, and vinegar (Siemen et al. 2018), leading to the question of how 5-KF is degraded in nature.

The analysis of hexose interconversion in *Erwinia* species showed that *Erwinia citreus* (now referred to as *T. citrea*) can use 5-KF, and possesses a NADPH-dependent reductase, which catalyzes the reduction of 5-KF to fructose (Schrimsher et al. 1988). Here we show that *T. morbirosei* possesses a very similar enzyme and can grow with 5-KF as carbon and energy source. In nature, 5-KF is produced by *Gluconobacter* species through oxidation of fructose by the membrane-bound enzyme Fdh (Ameyama et al. 1981; Kawai et al. 2013; Yamada et al. 1966; Siemen et al. 2018). The partial oxidation of sugars and polyols is a typical process for *Gluconobacter* species and is referred to as incomplete oxidation (Matsushita et al. 1994). Several sugars and polyols are attacked in this way. The partly oxidized products (e.g., ketogluconates from glucose and L-sorbose from D-sorbitol) are taken up by the cells and are consumed in the second growth phase or allow survival in the stationary phase (Matsushita et al. 1994). To channel the partially oxidized products into the central carbon metabolism, the organisms contain a repertoire of cytoplasmic oxidoreductases that reduce the products, which are then phosphorylated and degraded in the pentose phosphate pathway (Matsushita et al. 1994). The question was whether such a cytoplasmic oxidoreductase also exists for the reduction of 5-KF to fructose, which can be metabolized to gain ATP and intermediates for biosynthesis. Our growth experiments indicated that *Gluconobacter* species, which produce 5-KF naturally, were indeed able to utilize the sugar derivative as a carbon and energy source (Fig. 2).

*Tatumella* and *Gluconobacter* species are microorganisms thriving in aerobic habitats where 5-KF is commonly present due to the fructose dehydrogenase activity of certain *Gluconobacter* spp. Interestingly, we also found that the strictly fermentative anaerobe *C. pasteurianum* can grow with 5-KF as well, though other members of the order Clostridiales (e.g. *Clostridium leptum* and *Agathobacterobacter rectalis*) were not able to utilize this compound (Fig. S5). *C. pasteurianum* mainly uses mono- and polysaccharides as substrates, which are metabolized by glycolysis and converted to butyrate, acetate, and ethanol by butyrate fermentation. Although the organism does not share the same habitats as the strictly oxygen-dependent 5-KF producing *Gluconobacter* spp., it is not unlikely that *C. pasteurianum* is exposed to higher concentrations of 5-KF in nature. As an organism that was isolated from carrot slices and spoiled fruit juices (Dworkin and Gutnick 2012; Feng et al. 2010) *C. pasteurianum* is able to thrive in rotting fruit and vegetable matter, which presumably could have accumulated 5-KF beforehand due to the activity of *Gluconobacter* spp. Hence, it is tempting to speculate that the ability to metabolize 5-KF is not limited to 5-KF producing species but could also be found in bacteria that come in contact with this compound in their natural habitats.

In this context, it is important to understand whether the dietary consumption of 5-KF could also influence the human gut microbiota. As stated above, 5-KF is a potential candidate as an additive in food production for the reduction of high-calorie sugars in processed foods. It is therefore essential to investigate the influence of this compound on the human intestinal flora. The balance in the intestinal flora, which is closely linked to human health, can be shifted by diet. If microorganisms are able to metabolize 5-KF, unlike other representatives of the intestinal microbiota, they will be enriched while the latter group is suppressed. This may affect human well-being and digestion (Autenrieth 2017; Suez et al. 2014). We were able to show that representative microorganisms of the human intestinal tract could not grow on 5-KF as the sole carbon source. Because the selected microorganisms represented the most common and abundant genera in the human intestines, it is unlikely that metabolization of 5-KF occurs within the human gut in considerable amounts. Moreover, since there was also no production of fermentation end-products deriving from 5-KF like, e.g., the short-chain fatty acids propionate, acetate, or butyrate that could contribute to the nutritional value of the sugar derivative (Scheppach 1994), 5-KF can presumably be considered a calorie-free sugar substitute for human dietary applications.

Though we did not find any 5-KF reduction activity in the selected members of gut bacteria, the organisms we found capable of this catalysis were nevertheless phylogenetically rather distant. Unsurprisingly, this also led to the discovery of different enzymes that were responsible for the turnover of 5-KF. Altogether, we were able to identify four enzymes capable of 5-KF reduction that belong to three unrelated enzymatic classes with very different amino acid sequences and structural properties. The first one, TM-KFR from *T. morbirosei*, showed high similarities (94%) to the 5-KF reductase from *T. citrea*, which in turn is related to shikimate dehydrogenases (EC 1.1.1.25) (Schrimsher et al. 1988). In contrast, the second and third enzyme, GOX0644 from *G. oxydans* and CP-AKR from *C. pasteurianum*, belong to the aldo-keto reductase (AKR) superfamily, which show highest similarity to the 2,5-diketo-D-gluconate dehydrogenases from *E. coli* (70%) and *Corynebacterium* sp. (65%) together with the glyoxal reductase from *Bacillus subtilis* (69%). Lastly, protein GOX1432 from *G. oxydans* was previously described as a mannitol dehydrogenase (EC 1.1.1.138). The enzyme is responsible for mannitol-mediated osmoprotection in these organisms and reduces D-fructose to D-mannitol under physiological conditions (Zahid and Deppenmeier 2016). Furthermore, it has the ability to reduce L-sorbose to D-sorbitol and 5-ketogluconate to gluconate (Zahid and Deppenmeier 2016). Thus, protein GOX1432 shows a totally different substrate spectrum than described for KFR from *T. morbirosei* and *T. citrea*. As a basic principle, 5-KF reduction creates either L-sorbose or D-fructose depending on the stereoselectivity of the enzymes (Fig. 7). Our hypothesis is that the reduction of the *si* face of the planar C-carbonyl structure by the enzymes TM-KFR and GOX1432 leads to the formation of D-fructose. In the other case, the keto group is reduced at the *re* face by the 5-KF reducing enzymes GOX0644 and CP-AKR resulting in the production of L-sorbose.

## Supplementary Information

ESM 1 (PDF 786 kb)

## Data Availability

Data are available upon request from the authors.
