# Supplementary material for: Degradation of the low-calorie sugar substitute 5-ketofructose by different bacteria
Source: Appl Microbiol Biotechnol. 2021 Feb 22;105(6):2441–53. doi: 10.1007/s00253-021-11168-3 (PMC7954740; doi:10.1007/s00253-021-11168-3)
Supplement: Supplementary file 1 — (PDF 786 kb) [file 253_2021_11168_MOESM1_ESM.pdf]

Applied Microbiology and Biotechnology

Supplementary material

Degradation of the low-calorie sugar substitute 5-ketofructose by different bacteria

Jacqueline Schiessl[1], Konrad Kosciow[1], Laura S. Garschagen, Juliane J. Hoffmann, Julia Heymuth, Thomas Franke and Uwe Deppenmeier*

Institute of Microbiology and Biotechnology, University of Bonn, Meckenheimer Allee 168, 53115 Bonn, Germany

*For correspondence. E-mail: udeppen@uni-bonn.de; Tel. +49-228-735590; Fax +49-228-737576.

[1]Jacqueline Schiessl and Konrad Kosciow contributed equally to this work.

Tab. S1: Strains used for growth studies with 5-KF

Strain	Description	Source
Agathobacter rectalis DSM 17629	Wild-type strain	DSMZ (Braunschweig, Germany)
Alistipes shahii DSM 19121	Wild-type strain	DSMZ (Braunschweig, Germany)
Akkermansia muciniphila DSM 22959	Wild-type strain	DSMZ (Braunschweig, Germany)
Bifidobacterium adolescentis DSM 20083	Wild-type strain	DSMZ (Braunschweig, Germany)
Bacteroides vulgatus DSM 1447	Wild-type strain	DSMZ (Braunschweig, Germany)
Clostridium leptum DSM 753	Wild-type strain	DSMZ (Braunschweig, Germany)
Collinsella intestinalis DSM 13280	Wild-type strain	DSMZ (Braunschweig, Germany)
Dorea formicigenerans DSM 3992	Wild-type strain	DSMZ (Braunschweig, Germany)
Eubacterium siraeum DSM 15702	Wild-type strain	DSMZ (Braunschweig, Germany)
Escherichia coli K12 HB101	*supE*44, Δlac*U*169(Φ*lac*ZΔM15), *recA*1, *endA*1, *hsdR*17, thi-1, *gyrA*96, *relA*1	Bio-Rad (München, Germany)
Lactobacillus reuteri DSM 17509	Wild-type strain	DSMZ (Braunschweig, Germany)
Methanomassiliicoccus luminyensis DSM 25720	Wild-type strain	DSMZ (Braunschweig, Germany)
Prevotella copri DSM 18205	Wild-type strain	DSMZ (Braunschweig, Germany)
Parabacteroides johnsonii DSM 18315	Wild-type strain	DSMZ (Braunschweig, Germany)
Ruminicoccus gnavus DSM 108212	Wild-type strain	DSMZ (Braunschweig, Germany)

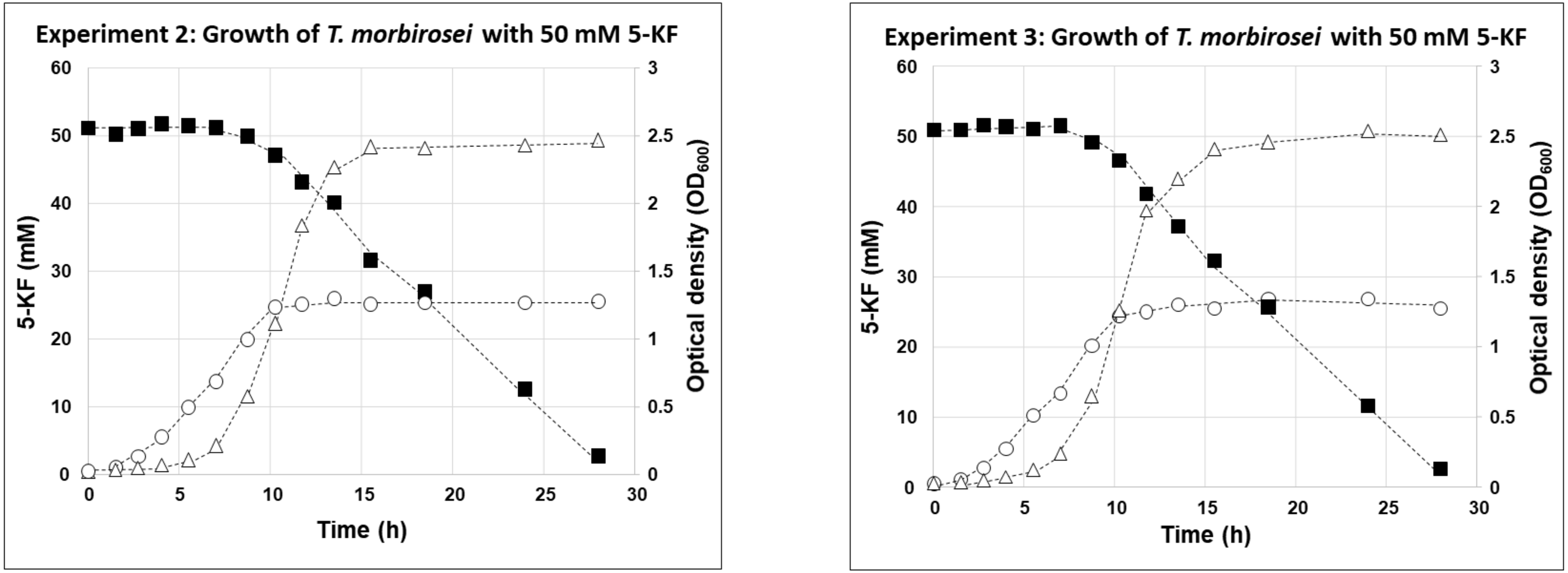

Fig. S1: Growth and 5-KF consumption in *T. morbirosei*. Cells were grown in 0.6 % yeast extract with 50 mM 5-KF or without 5-KF at 30°C and 180 rpm. (■) 5-KF concentration, (Δ) OD_{600} of 5-KF containing cultures, (○) OD_{600} of control cultures without 5-KF. For the quantification of 5-KF, 1 ml samples were taken from the cultures at different time points and centrifuged at 13,000 × *g* for 1 min. The detection of 5-KF was performed by HPLC (Knauer Smartline HPLC system, Knauer GmbH, Berlin, Germany) with an Aminex HPX-87H column (BioRad, Munich, Germany, 300 mm × 7.8 mm) using 5 mM H_2SO_4 as mobile phase at a column temperature of 65 °C and a flow rate of 0.6 ml per min. The substrate was quantified by a refraction index detector (RI detector; Azura RID2.1 L, Knauer GmbH, Berlin, Germany) and a UV detector (Smartline 2600, Knauer, Berlin, Germany) at 210 nm by comparison to a calibration curve. Experiment 1 is shown in the main text.

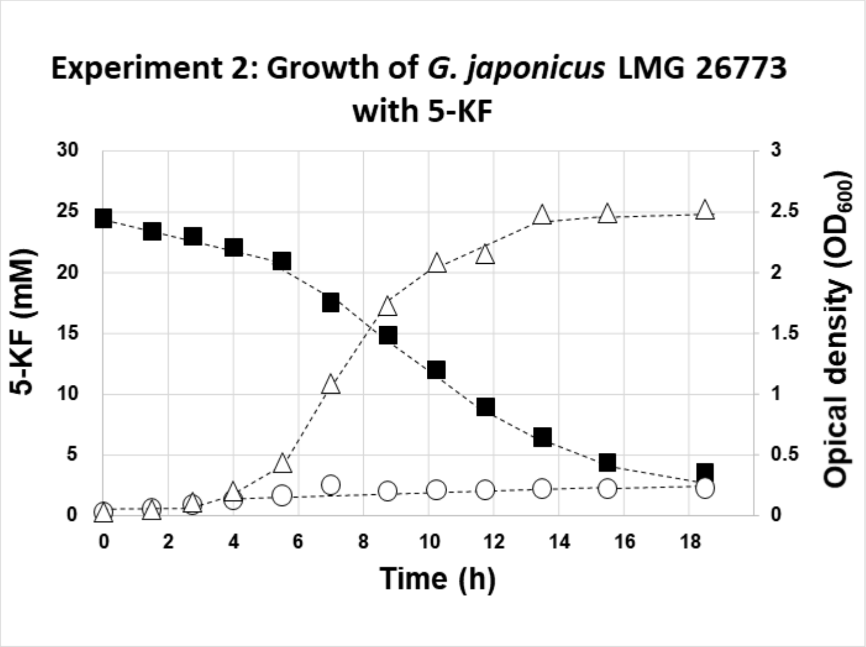

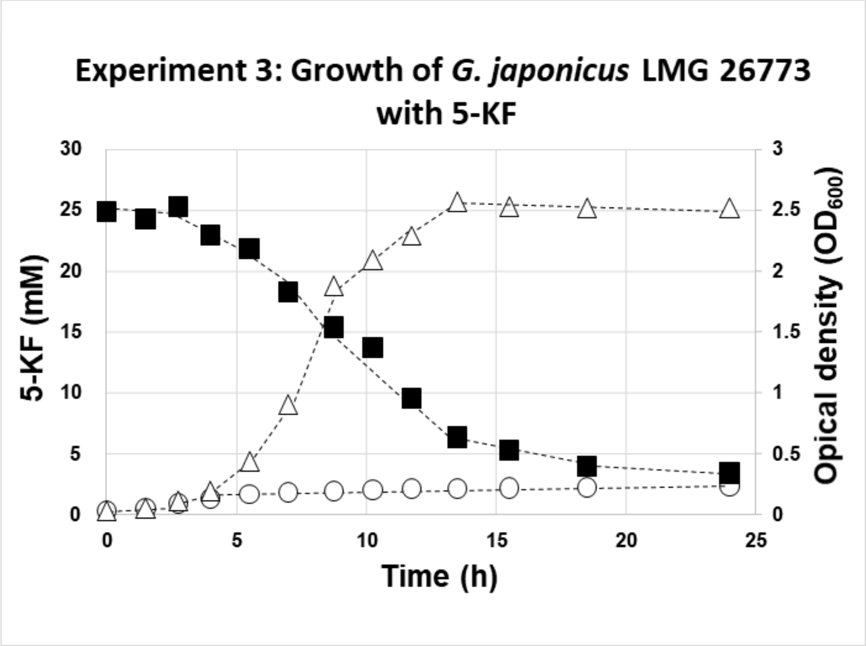

Fig. S2: Growth of *G. japonicus* LMG 26773 on 5-KF. (■) 5-KF concentration, (Δ) OD_{600} in the presence of 5-KF, (○) OD_{600} in the absence of 5-KF. *G. japonicus* LMG 26773 was cultivated in 100 ml YKF-medium containing 25 mM 5-KF and 0.6 % (w/v) yeast extract at 30°C and 180 rpm. Negative control cultures were grown in Y-Medium composed of 0.4 % (w/v) yeast extract. The quantification of 5-KF is described in Fig. S1. Experiment 1 is shown in the main text.

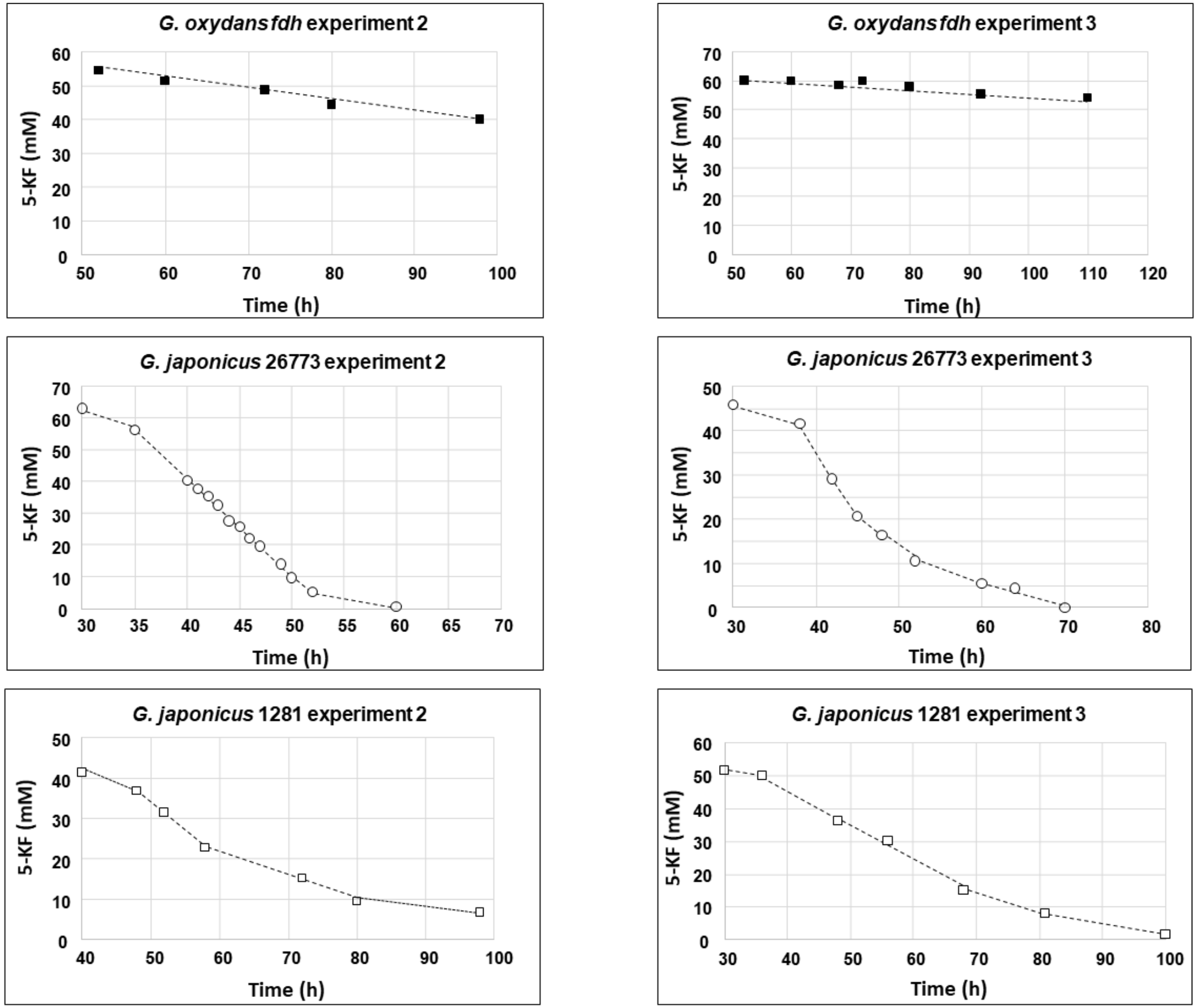

Fig. S3: Degradation of 5-KF in the stationary growth phase by different *Gluconobacter* strains grown on fructose as substrate. (■) *G. oxydans fdh*, (□) *G. japonicus* LMG 1281, (○) *G. japonicus* LMG 26773. All strains were cultivated in 100 ml YF-medium containing 100 mM fructose, 0.6% (w/v) yeast extract and 50 µg/ml cefoxitin at 30°C and 180 rpm. The decrease of 5-KF was determined in the stationary growth phase (≥ 30 h). The quantification of 5-KF is described in Fig. S1. Experiment 1 is shown in the main text.

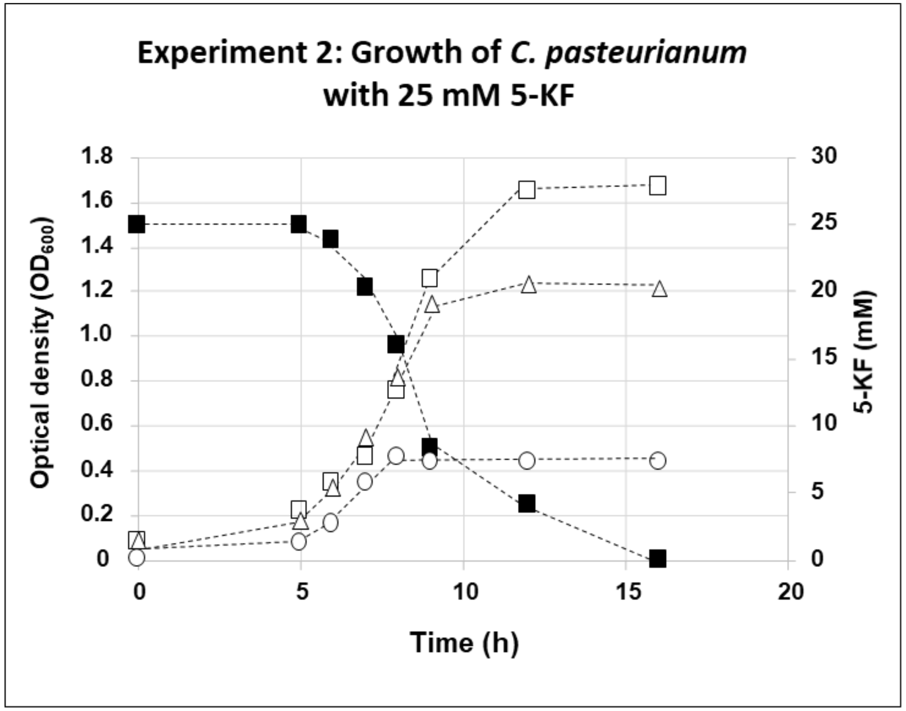

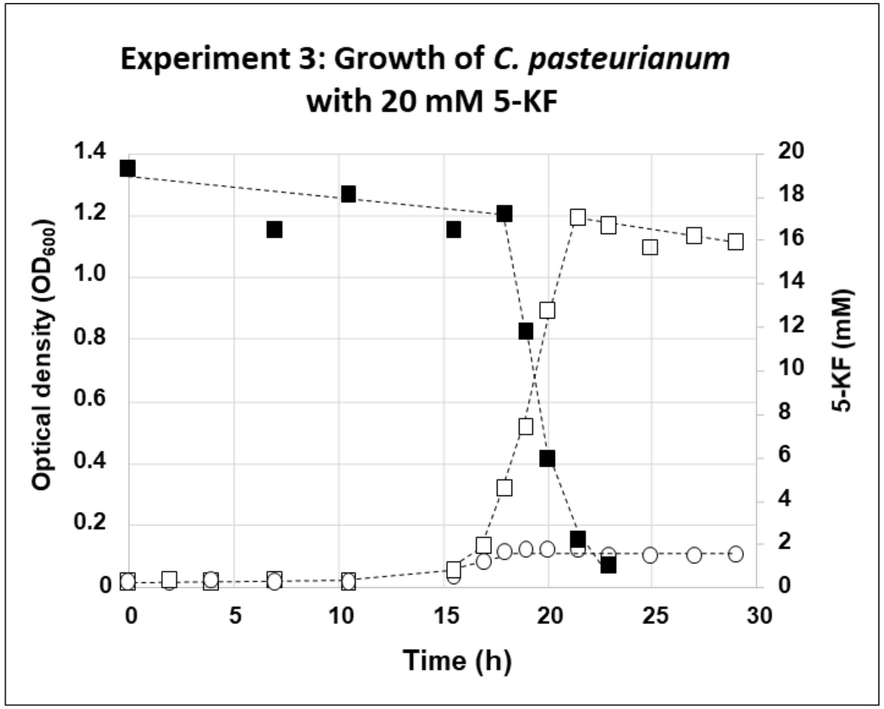

Fig. S4 Growth and 5-KF consumption in *C. pasteurianum.* Cells were grown at 37 ºC in 0.4 % yeast extract medium supplemented with 48 mM $NaHCO_3$, modified mineral 3B solution, 0.5 g/L cysteine, and 25 mM 5-KF or 25 mM glucose (experiment 2). The same conditions were applied in experiment 3 with the exception that only 20 mM 5-KF was added. (■) 5-KF concentration, (Δ) OD_{600} of 5-KF containing cultures, (○) OD_{600} of control cultures without carbon source, (□) OD_{600} of glucose grown cultures (experiment 2). Experiment 1 is shown in the main test. The detection of substrates was performed by HPLC (Knauer Smartline HPLC system, Knauer GmbH, Berlin, Germany) with an Aminex HPX-87H column (BioRad, Munich, Germany, 300 mm × 7.8 mm) using 5 mM H_2SO_4 as mobile phase at a column temperature of 65 °C and a flow rate of 0.6 ml per min. The substrates were quantified by a refraction index detector (RI detector; Azura RID2.1 L, Knauer GmbH, Berlin, Germany) and a UV detector (Smartline 2600, Knauer, Berlin, Germany) at 210 nm by comparison to calibration curves.

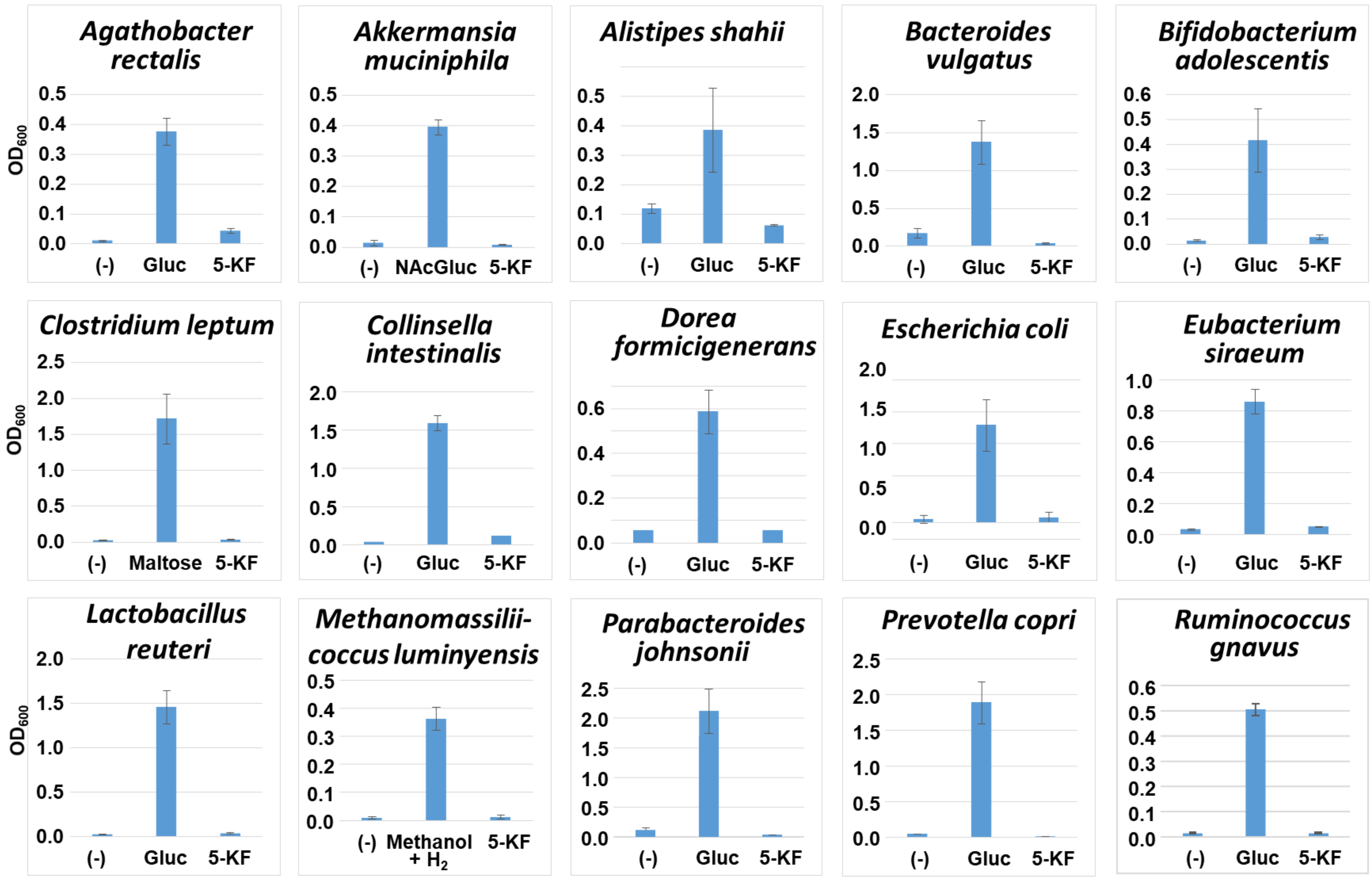
Agathobacter rectalis
OD600
0.5
0.4
0.3
0.2
0.1
0.0
(-)
Gluc
5-KF
Akkermansia muciniphila
0.5
0.4
0.3
0.2
0.1
0.0
(-)
NAcGluc
5-KF
Alistipes shahii
0.5
0.4
0.3
0.2
0.1
0.0
(-)
Gluc
5-KF
Bacteroides vulgatus
2.0
1.5
1.0
0.5
0.0
(-)
Gluc
5-KF
Bifidobacterium adolescentis
0.6
0.5
0.4
0.3
0.2
0.1
0.0
(-)
Gluc
5-KF
Clostridium leptum
OD600
2.5
2.0
1.5
1.0
0.5
0.0
(-)
Maltose
5-KF
Collinsella intestinalis
2.0
1.5
1.0
0.5
0.0
(-)
Gluc
5-KF
Dorea formicigenerans
0.6
0.4
0.2
0.0
(-)
Gluc
5-KF
Escherichia coli
2.0
1.5
1.0
0.5
0.0
(-)
Gluc
5-KF
Eubacterium siraeum
1.0
0.8
0.6
0.4
0.2
0.0
(-)
Gluc
5-KF
Lactobacillus reuteri
OD600
2.0
1.5
1.0
0.5
0.0
(-)
Gluc
5-KF
Methanomassilii-coccus luminyensis
0.5
0.4
0.3
0.2
0.1
0.0
(-)
Methanol + H2
5-KF
Parabacteroides johnsonii
2.5
2.0
1.5
1.0
0.5
0.0
(-)
Gluc
5-KF
Prevotella copri
2.5
2.0
1.5
1.0
0.5
0.0
(-)
Gluc
5-KF
Ruminococcus gnavus
0.6
0.5
0.4
0.3
0.2
0.1
0.0
(-)
Gluc
5-KF

Fig. S5: Growth experiments with 5-KF using representative members of the human gut microbiota. (-), growth without carbon source; Gluc, glucose; NAcGluc, N-acetylglucosamin; 5-KF, 5-keto-D-fructose.

Akkermansia muciniphila, *Agathobacter rectalis*, *Alistipes shahii*, *Bacteroides vulgatus*, *Prevotella copri*, *Bifidobacterium adolescentis*, *Clostridium leptum*, *Collinsella intestinalis*, *Escherichia coli*, *Eubacterium siraeum*, *Lactobacillus reuteri*, *Parabacteroides johnsonii* and *Ruminococcus gnavus* were grown in YM medium composed of 0.4 % yeast extract and 5 % modified mineral 3B solution (40 mM KH_2PO_4, 300 mM NaCl, 4 mM $CaCl_2$ x 2 H_20, 2 mM $MgCl_2$ x 6 H_20, 1 mM $MnCl_2$ x 4 H_20, 0.1 mM $CoCl_2$ x 6 H_20, 35 mM Na_2SO_4 (Varel and Bryant 1974)) in serum flasks sealed with butyl rubber stoppers under a N_2/CO_2 atmosphere (80/20%). Substrates were added to a final concentration of 20 mM as indicated (Gluc, glucose; NAcGluc, N-acetylglucosamin). Prior to inoculation, L-cysteine (0.5 g/l) as reducing agent, vitamin K1 (0.1% (v/v)) and hemin (5 mg/l) were added. The medium of *Akkermansia muciniphila* and *Lactobacillus reuteri* was additionally supplemented with 4 mM L-threonine and 60 mM Na-acetate, respectively. The pH value was adjusted to 7.0 using a $NaHCO_3$ solution. *Dorea formicigenerans* was grown on PY medium (Dowell and Hawkins 1974), pH 7, in serum flasks under a N_2/CO_2 (80/20) atmosphere. *Methanomassiliicoccus luminyensis* was cultivated according to Dridi *et al.* (2012) with 75 mM methanol and 1 bar H_2/CO_2. OD_{600} was measured after 24 h. All organisms were cultivated at 37 °C.

All strains grew with their preferred substrates to an optical density of 0.4 to 1.8. Final optical densities (OD_{600}) with 5-KF as substrate were low and comparable to cultures without an additional carbon source. HPLC analysis revealed that 5-KF was not consumed, indicating that the sugar derivative was not used as carbon and energy source.

References:

Dowell VR, Hawkins TM (1974) Laboratory methods in anaerobic bacteriology, CDC Laboratory Manual. CDC Information Center, Georgia

Dridi B, Fardeau ML, Ollivier B, Raoult D & Drancourt M (2012) *Methanomassiliicoccus luminyensis* gen. nov., sp. nov., a methanogenic archaeon isolated from human faeces. Int J Syst Evol Microbiol 62: 1902–1907. doi: 10.1099/ijs.0.033712-0

Varel VH, Bryant MP (1974) Nutritional features of *Bacteroides fragilis* subsp. *fragilis*. Appl Microbiol 2: 251–257. doi: 10.1128/aem.28.2.251-257.1974

```
Gja0644     MIDPVAYMDNQTRITLNDGCIIPQLGLGVWKTPPEETAHVVREAIKLGYHSVDTARLYQN     60
Gox0644     MSSQVPSAEAQTVISFHDGHTMPQIGLGVWETPPDETAEVVKEAVKLGYRSVDTARLYKN     60
            * . *   : ** *:::**  :**:*****:***:***.**:**:****:********:*

Gja0644     EAGVGEGLAGSPDVFVTTKVWNDEQGYDSTIRAYEESCRLLKRPVLDMYLIHWPMPEQGQ     120
Gox0644     EEGVGKGLEDHPEIFLTTKLWNDEQGYDSTLRAYEESARLLRRPVLDLYLIHWPMPAQGQ     120
            * ***:** . *::*:***:**********:******.***:*****:******** ***

Gja0644     YVETWKALVDLQKDGRVKSIGVSNFEPEHLERIMDATGVVPAVNQIELHPFFQQEKVRAF     180
Gox0644     YVETWKALVELKKSGRVKSIGVSNFESEHLERIMDATGVVPVVNQIELHPDFQQRALREF     180
            *********:*:*.************ **************.******** ***. :* *

Gja0644     NEQHNIRTEAWRPLGKGQLLDNATIGAIARHVGRTPAQVIIRWHLQSGFIVIPKSANLKR     240
Gox0644     HEKHNIRTESWRPLGKGRVLSDERIGKIAEKHSRTPAQVVIRWHLQNGLIVIPKSVNPKR     240
            :*:******:*******::*.:  ** **.: .******:******.*:******.* **

Gja0644     LAENLDVFDFSLDDADMAAITALDREDGRMGAHPMTARF   279
Gox0644     LAENLDVFGFVLDADDMQAIEQMDRKDGRMGADPNTAKF   279
            ********.* **  ** **  :**:******.* **:*
```

```
Gja1432      MITHEILKSLPVGVQAPPYDINGIKPGIVHFGVGNFFRAHEAFYVEQILKDDPNWGIIGV     60
Gox1432      MITRETLKSLPANVQAPPYDIDGIKPGIVHFGVGNFFRAHEAFYVEQILEHAPDWAIVGV     60
             ***:* *****..********:***************************:. *:*.*:**
Gja1432      GLTGSDRSKKKAEEFKKQDCLFSLTETAPSGKSTVRVMGALRDYLLAPADPEAVLKHLAD     120
Gox1432      GLTGSDRSKKKAEEFKAQDCLYSLTETAPSGKSTVRVMGALRDYLLAPADPEAVLKHLVD     120
             **************** ****:************************************.*
Gja1432      PAIRIVSMTITEGGYNINETTGEFDLQNKAVQQDLKTPETPSTIFGYVVEGLRRRRDAGG     180
Gox1432      PAIRIVSMTITEGGYNINETTGAFDLENAAVKADLQNPEKPSTVFGYVVEALRRRRDAGG     180
             ********************** ***:* **: **:.**.***:******.*********
Gja1432      KAFTIMSCDNLRHNGNVARKAFLGYAKARDPELAKWIEENATFPNGMVDRITPTVSADIA     240
Gox1432      KAFTVMSCDNLRHNGNVARKAFLGYAKARDPELAKWIEENATFPNGMVDRITPTVSAEIA     240
             ****:****************************************************:**
Gja1432      KKLNDASGLNDDLPLVAEDFHQWVLEDNFADGRPALEKAGVQFVEDVTDYEHVKIRMLNA     300
Gox1432      KKLNAASGLDDDLPLVAEDFHQWVLEDRFANGRPPLEKAGVQLVDDVTDWEHVKIRMLNA     300
             **** ****:*****************.**:*** *******:*:****:**********
Gja1432      GHIMLCFPGVLAGYENVDQALADADLRRNLENFLNKDVIPTLEAPPGMTLEGYRDSVISR     360
Gox1432      GHITLCFPGILVGYENVDDAIEDKDLRGNLENYLNKDVIPTLKAPPGMTLEGYRDSVISR     360
             *** *****:*.******:*: * *** ****:*********:*****************
Gja1432      FSNPAMADQTLRISGDGSSKIQVFWTETVRQALEGKRDLSRIAFGMASYLEMLRGKDEKG     420
Gox1432      FSNKAMSDQTLRIASDGCSKIQVFWTETVRRAIECKRDLSRIAFGIASYLEMLRGRDEKG     420
```

```
              *** **:******:.**.************:*:* **********:*********:****
Gja1432       DTYEPFEPTFDESQKILAKADDFESALKLPAFDAWSDLDTSELNGKVVALRKIIREKGVK     480
Gox1432       GTYESSEPTYGEAQKKLAKADDFESALKLPAFDGWRDLDTSELDQKVIALRKVIREKGVK     480
              .***  ***:.*:** ****************.* *******: **:****:*******
Gja1432       AALPA      485
Gox1432       AAIPA      485
              **:**
```

Fig. S6: Alignment of 5-KF reductases from *G. japonicus* LMG 26773 with their counterparts from *G. oxydans* 621H

The amino acid sequence alignment was performed with the program Clustal Omega (https://www.ebi.ac.uk/Tools/msa/clustalo/) using default parameters.

Amino acid sequences: Gja0644 (KXV40663.1) from *G. japonicus* LMG26773:
MIDPVAYMDNQTRITLNDGCIIPQLGLGVWKTPPEETAHVVREAIKLGYHSVDTARLYQNEAGVGEGLAGSPDVFVTTKVWNDEQGYDSTIRAYEESCRLLKRPVL
DMYLIHWPMPEQGQYVETWKALVDLQKDGRVKSIGVSNFEPEHLERIMDATGVVPAVNQIELHPFFQQEKVRAFNEQHNIRTEAWRPLGKGQLLDNATIGAIARH
VGRTPAQVIIRWHLQSGFIVIPKSANLKRLAENLDVFDFSLDDADMAAITALDREDGRMGAHPMTARF
Gox0644 from *G. oxydans* 621H
MSSQVPSAEAQTVISFHDGHTMPQIGLGVWETPPDETAEVVKEAVKLGYRSVDTARLYKNEEGVGKGLEDHPEIFLTTKLWNDEQGYDSTLRAYEESARLLRRPVL
DLYLIHWPMPAQGQYVETWKALVELKKSGRVKSIGVSNFESEHLERIMDATGVVPVVNQIELHPDFQQRALREFHEKHNIRTESWRPLGKGRVLSDERIGKIAEKH
SRTPAQVVIRWHLQNGLIVIPKSVNPKRLAENLDVFGFVLDADDMQAIEQMDRKDGRMGADPNTAKF
Gja1432 (KXV41067.1) from *G. japonicus* LMG26773
MITHEILKSLPVGVQAPPYDINGIKPGIVHFGVGNFFRAHEAFYVEQILKDDPNWGIIGVGLTGSDRSKKKAEEFKKQDCLFSLTETAPSGKSTVRVMGALRDYLLAP
ADPEAVLKHLADPAIRIVSMTITEGGYNINETTGEFDLQNKAVQQDLKTPETPSTIFGYVVEGLRRRRDAGGKAFTIMSCDNLRHNGNVARKAFLGYAKARDPELA
KWIEENATFPNGMVDRITPTVSADIAKKLNDASGLNDDLPLVAEDFHQWVLEDNFADGRPALEKAGVQFVEDVTDYEHVKIRMLNAGHIMLCFPGVLAGYENVD
QALADADLRRNLENFLNKDVIPTLEAPPGMTLEGYRDSVISRFSNPAMADQTLRISGDGSSKIQVFWTETVRQALEGKRDLSRIAFGMASYLEMLRGKDEKGDTYE
PFEPTFDESQKILAKADDFESALKLPAFDAWSDLDTSELNGKVVALRKIIREKGVKAALPA
Gox1432 from *G. oxydans* 621H
MITRETLKSLPANVQAPPYDIDGIKPGIVHFGVGNFFRAHEAFYVEQILEHAPDWAIVGVGLTGSDRSKKKAEEFKAQDCLYSLTETAPSGKSTVRVMGALRDYLLA
PADPEAVLKHLVDPAIRIVSMTITEGGYNINETTGAFDLENAAVKADLQNPEKPSTVFGYVVEALRRRRDAGGKAFTVMSCDNLRHNGNVARKAFLGYAKARDPE
LAKWIEENATFPNGMVDRITPTVSAEIAKKLNAASGLDDDLPLVAEDFHQWVLEDRFANGRPPLEKAGVQLVDDVTDWEHVKIRMLNAGHITLCFPGILVGYENV
DDAIEDKDLRGNLENYLNKDVIPTLKAPPGMTLEGYRDSVISRFSNKAMSDQTLRIASDGCSKIQVFWTETVRRAIECKRDLSRIAFGIASYLEMLRGRDEKGGTYES
SEPTYGEAQKKLAKADDFESALKLPAFDGWRDLDTSELDQKVIALRKVIREKGVKAAIPA
